# Automatic Plant Disease Detection Based on Tranvolution Detection Network With GAN Modules Using Leaf Images

**DOI:** 10.3389/fpls.2022.875693

**Published:** 2022-05-26

**Authors:** Yan Zhang, Shiyun Wa, Longxiang Zhang, Chunli Lv

**Affiliations:** ^1^College of Information and Electrical Engineering, China Agricultural University, Beijing, China; ^2^College of Science, China Agricultural University, Beijing, China

**Keywords:** transformer, Generative Adversarial Networks, detection network, deep learning, plant disease detection, leaf images

## Abstract

The detection of plant disease is of vital importance in practical agricultural production. It scrutinizes the plant's growth and health condition and guarantees the regular operation and harvest of the agricultural planting to proceed successfully. In recent decades, the maturation of computer vision technology has provided more possibilities for implementing plant disease detection. Nonetheless, detecting plant diseases is typically hindered by factors such as variations in the illuminance and weather when capturing images and the number of leaves or organs containing diseases in one image. Meanwhile, traditional deep learning-based algorithms attain multiple deficiencies in the area of this research: (1) Training models necessitate a significant investment in hardware and a large amount of data. (2) Due to their slow inference speed, models are tough to acclimate to practical production. (3) Models are unable to generalize well enough. Provided these impediments, this study suggested a Tranvolution detection network with GAN modules for plant disease detection. Foremost, a generative model was added ahead of the backbone, and GAN models were added to the attention extraction module to construct GAN modules. Afterward, the Transformer was modified and incorporated with the CNN, and then we suggested the Tranvolution architecture. Eventually, we validated the performance of different generative models' combinations. Experimental outcomes demonstrated that the proposed method satisfyingly achieved 51.7% (*Precision*), 48.1% (*Recall*), and 50.3% (*mAP*), respectively. Furthermore, the SAGAN model was the best in the attention extraction module, while WGAN performed best in image augmentation. Additionally, we deployed the proposed model on Hbird E203 and devised an intelligent agricultural robot to put the model into practical agricultural use.

## 1. Introduction

Agriculture is a milestone and a booster of early human social development. The substantial advances in science and technology brought about by the prosperity of human society have been assisting in developing agriculture. In recent decades, modern technology has empowered humans to produce enough food to feed seven billion people (Mohanty et al., 2016). However, many developing countries face a food crisis (Erokhin and Gao, 2020), suffering from famine and economic loss. Although political factors in developing countries, such as social unrest and economic instability, indeed affect the production and distribution of food, it is undeniable that those countries lack cutting-edge science and technology in agriculture. Moreover, food security is typically threatened by diverse objective aspects, including climate change (Anderson et al., 2020), plant pests and diseases (Trebicki and Finlay, 2019), and others. In addition, at the stage of plant's leaf quality testing, underdeveloped regions rely mainly on the workforce to perform leaf classification in terms of quality or omit this process due to high cost. This decision leads to stagnation of the agricultural economy in these regions and further constrains the improvement of the local population's living standard. Therefore, developing a positively automated and low-cost leaf disease detection method is imperative.

Object detection of plant diseases retains a wide range of application prospects in agriculture, providing timely feedback on plant conditions, guiding crop cultivation and post-treatment, and thus significantly declining costs. Thanks to the blossoming of microcomputers and mobile computing devices, hardware support has been supplied for plant object detection. Meanwhile, backpropagation algorithms-based deep learning methods (especially convolutional neural networks) provide software support. Accordingly, several researchers have initiated developing automatic deep learning-based algorithms for plant disease detection applications.

Mohanty et al. (2016) trained a deep convolutional neural network(CNN) to recognize 14 crops and 26 diseases (with or without disease). The trained model achieved 99.35% accuracy on a reserved test set, reflecting the viability of deep learning in crop detection. More specifically, Ramcharan et al. (2017) suggested a deep CNN-based disease detection method, attempting to deploy the program to mobile devices. Liu and Wang (2020) improved the existing technique of tomato pest image recognition based on the YOLO-v3 model (an efficient object detection algorithm based on CNNs), improve the existing technique of tomato pest image recognition in the natural. Xu et al. (2022) provided an approach for data augmentation that can fully utilize data from non-target regions of sample images to optimize deep learning models for disease detection. Their method is more applicable to plant disease detection than common data enhancement approaches. Zhang et al. (2021b) employed an enhanced CNN model to detect pear flaws; more precisely, the defect images were expanded by a deep convolutional adversarial generation network (DCGAN). On the three thousand validation set, the detection accuracy reached 97.35% exactly. Besides, various mainstream CNNs were compared to thoroughly evaluate the performance of models. Subsequently, the top performed one was chosen to conduct additional comparative experiments using traditional machine learning approaches. Agarwal et al. (2020) suggested a CNN-based approach to detect tomato leaf diseases. They conceived three convolution-pooling layers and two fully connected layers. Experimentally, the efficacy of the presented model outstripped the pre-trained model, namely, MobileNet, InceptionV3, and VGG16. The classification accuracy fluctuated between 76 and 100%, and the offered model's average accuracy was 91.2% for nine diseases and one healthy category. Pantazi et al. (2019) utilized one-class classification and local binary patterns (LBPs) to demonstrate an automated strategy for identifying crop diseases on multiple leaf images matching diverse crop species. The suggested methodology employs a separate one-class classifier for each plant health state. They tested the algorithms developed on vine leaves in various plants, finding them highly applicable when applied to other plants. The 46 plant-condition combinations reached an entire success rate of 95%. A multi-activation function (MAF) module was suggested to improve the CNN by Zhang et al. (2021a). The diseased samples were expanded and supplemented using image preprocessing measures, and the training speed was raised using transfer learning and warm-up approaches. The proposed system could efficiently and correctly detect three types of maize diseases, achieving a 97.41% accuracy rate in the validation set, exceeding conventional artificial intelligence methods.

Although deep learning has contributed to considerable progress in plant disease detection, traditional algorithms have remained the following obstacles that are not neglectable.

1. High model training costs require prodigious amounts of data and expensive hardware costs and are challenging to deploy to mobile devices.
2. The inference speed of successfully trained models is relatively slow, and thus, those models are tricky to be adapted to practical production.
3. The generalization capability of the models is unsatisfactory, and a lack of equally effective models for different plant leaves cannot be overlooked.

Driven by the above impediments, previous studies, and the vital significance of enhancing the efficiency of object detection on leaf diseases, this article proposed a high-performance network for leaf image detection. The network incorporates the CNN and transformer with GAN modules to improve mainstream detection networks. This study's primary innovations are as follows:

1. We adjusted the transformer to decrease the number of parameters, accelerating the training and working as a branch network to improve CNN's global feature extraction ability. Tranvolution's performance is superior to CNN and ViT with comparable parameter complexity as it inherits and integrates the structural and global feature extraction benefits of CNN and visual transformers. It has proved its extraordinary potential in leaf image detection. Ultimately, the offered technique achieved 51.7% (*Precision*), 48.1% (*Recall*), and 50.3% (*mAP*) on the validation set. According to this experimental outcome, the suggested model surpasses all comparable models.
2. Given the complexity of the dataset utilized in this article, various data enhancement methods were employed. Additionally, this study suggested an original pre-processing method to remove the leaf vein details for the leaf images.
3. This article encapsulated the proposed model and optimized the matrix multiplication at the instruction level to run efficiently on the Hbird E203 CPU. In addition, an intelligent agricultural robot was built based on this hardware platform, allowing the application of the model in real-world agricultural scenes.

The remaining sections of this study are organized as follows: Section 2 briefly describes the object detection network's development and its recent achievements. Section 3 introduces the dataset utilized in this article. Section 4 explicitly describes the Tranvolution detection network with GAN modules. Section 5 defines the experimental setup and evaluation metrics. Section 6 demonstrates and analyzes detection and validation results. Section 7 operates multiple ablation experiments to prove the optimized method's efficiency and discusses the proposed method's limitations. Section 8 is a summary of the entire article.

## 2. Related Study

Object detection is a vibrant topic in computer vision research and is a crucial visual content analysis and comprehension. The benefits in applications such as autonomous vehicles and medical diagnosis brought by object detection are noticeable. This section will expound on deep learning techniques applied to object detection.

Scientists have recognized that CNNs offer an efficient framework for processing images as a result of AlexNet's superb work on ImageNet (Krizhevsky et al., 2012). CNNs are used in a variety of computer vision tasks due to their flexibility. Object detection is one of these tasks, whose algorithms can be classified into anchor-based and anchor-free categories.

### 2.1. Anchor-Based Object Detection Algorithms

Anchor-based algorithms have two types: two-stage algorithms and one-stage algorithms. Typically, two-stage algorithms are more accurate, whereas one-stage algorithms are faster.

#### 2.1.1. Two-Stage Algorithms

The following shows the steps of two-stage algorithms:

**Stage 1**: Yield regional suggestions from images.

**Stage 2**: Create ultimate object edges from region suggestions.

In 2014, Girshick et al. (2014) and Girshick (2015) developed R-CNN, which used the selective search algorithm to choose potential object frames from a group of object candidate frames. Images in these selected frames were then resized to a certain fixed size and sent to an ImageNet-trained CNN model. Afterward, extracted features were sent into a classifier, which predicted if a target would be detected in that object frame and, if so, to whose category it belongs. Despite the fact that the R-CNN algorithm has progressed significantly, the computation of overlapping frames was too redundant, which declined the entire network's detection speed. Therefore, to decrease this kind of unnecessary computation, He et al. (2015) proposed SPP-Net underlying a distinctive shape, Spatial Pyramid Pooling Layer (SPP). SPP-Net partitioned an image into diverse blocks, such as 1, 4, or 8 blocks, and then fused the extracted features of each block to account for features in different scales. When applying this network to detect objects, the full image is just computed once to build the corresponding feature map, avoiding the redundant convolutional feature map computations. For classification, SPP-Net used support vector machines (SVMs), which have a high storage space demand, and merely train the model for the fully connected layer.

Girshick et al. (2014) and Girshick (2015) improved R-CNN and SPP-Net and then released Fast R-CNN in 2015. Fast R-CNN began with an input image fed to the CNN for feature extraction and returning prospective region ROIs. Subsequently, the ROIs were subjected to an ROI pooling layer to guarantee the same size of each region. Finally, the fully connected layer received these regions' features for classification. Even though Fast R-CNN processes an image in 2 s (compared to 14 s for R-CNN), it is still too slow to be applied in practical production. For employing CNN models to directly create candidate frames, Ren et al. (2015) initiated Faster R-CNN—an end-to-end, closest-to-real-time performance deep learning detection network. The main contribution of this algorithm was the proposal of a region selection network for creating candidate frames, which considerably improved detection frame generation speed. Inspired by the Faster R-CNN, Lin et al. (2017) proposed a feature pyramid network (FPN) in 2017. FPN presented a top-down network structure with lateral connections to construct high-level semantic information. It vastly increased the accuracy of the detection network, particularly for datasets containing huge-scale variations of the objects.

#### 2.1.2. One-Stage Algorithms

YOLO-v1 (Redmon et al., 2016), the first one-stage deep learning detection algorithm, divides an image into several grids, then predicts the bounding box for each grid at the same time and provides the associated probability. Though YOLO-v1 is significantly faster than two-stage algorithms, it is less accurate, particularly on small objects. The single shot multibox detector (SSD) algorithm was then proposed by Liu et al. (2016). The suggested multi-resolution and multi-reference detection approaches were the algorithm's main innovations. The SSD technique differs from partial earlier detection algorithms in that some detection algorithms solely detect at the network's deepest branch. SSD, on the contrary, possesses several detection branches that can recognize objects of various sizes. Accordingly, SSD significantly enhances multi-scale object detection accuracy and is considerably more feasible for small object detection. YOLO-v4 (Bochkovskiy et al., 2020) is the YOLO algorithm's fourth iteration. To be more specific, (1) on the input side, mosaic data augmentation, cross mini-batch normalization (CmBN), and self-adversarial training (SAT) are employed. (2) YOLO-v4 introduces new methods on the feature extraction network, such as dropblock, mish activation function, and CSPDarknet53. (3) The SPP module is integrated into the detection head. In conclusion, YOLO-v4 has prominent significance in engineering, as it introduced the most recent research attainments in deep learning and realized a considerable leap forward from YOLO-v3.

The anchor-based object detection algorithms have the following four drawbacks:

1. Anchor size, number, and shape significantly impact detection performance (by varying these hyperparameters, Retinanet improves AP by 4% on the COCO benchmark), so anchor-based detection performance is susceptible to the size, number, and shape of the anchors.
2. These fixed anchors considerably compromise the generalization capability of the detector, resulting in the anchor having to be resized and reshaped for different tasks.
3. To match the actual frame, numerous anchors need to be generated. Nonetheless, most of the anchors are marked as negative samples during training, so it causes the issue of extreme sample imbalance.
4. In training, the network needs to calculate the IOU of each anchor with the actual frame, which consumes plenty of memory and time.

### 2.2. Anchor Free Object Detection Algorithms

Anchor-based detection algorithms are computationally complicated due to abundant anchors, and multiple hyperparameters can affect model performance. The recent anchor-free technique discards anchors and accomplishes detection by identifying key points, which considerably declines the number of hyperparameters.

CornerNet is the pioneer of the anchor-free technical route, which proposes a new object detection method—transform the detection of the target bounding box by the network into the detection of a pair of key points (i.e., the lower right and upper left corners), by detecting objects as pairs of key points without designing an anchor box as a priority. However, CornerNet focuses solely on edges and corner points and lacks information about the target's interior. Unlike CornerNet, the structure of CenterNet (Duan et al., 2019) is straightforward. It abandons the idea of two critical points in the lower right and upper left corners but directly detects the target's center point. Furthermore, other features such as 3D position, size, orientation, and even pose can be regressed using the image features at the center point location, which is truly anchor-free. Nevertheless, it is straightforward to overlap the prediction results when two similar objects are in close proximity within the image sample. FSAF (Zhu et al., 2019) proposed a module for training the anchor free branch in the feature pyramid, allowing each object to automatically select the most appropriate feature. In this module, the size of the anchor box no longer determines which features are selected for prediction, making the size of the anchor an irrelevant variable and automating the model to learn to select features. The FCOS (Tian et al., 2019) network is a pixel-by-pixel target detection algorithm based on FCN, which implements the solution of anchor free and proposal free and proposes the idea of center ness. The algorithm avoids complex operations by removing the anchor, saves a large amount of memory occupation during training, and reduces the total training memory occupation space by about two times.

### 2.3. Transformer Architecture

Transformer architecture for vision tasks has recently been presented. Visual transformer (ViT) (Han et al., 2020; Sajid et al., 2021; Truong et al., 2021) establish the possibility of pure transformer architectures for computer vision tasks as a pioneering study. Transformer blocks are utilized as standalone architectures or presented into CNNs for semantic segmentation, image classification, image generation, image enhancement, and object detection to manipulate long-range dependencies. On the other hand, the visual transformers' particular self-attentive mechanism frequently overlooks local features. Furthermore, transformers typically surpass CNNs in terms of all-around performance on massive datasets. Provided the situations mentioned above—the vast and irreparable deficit of transformer architectures—this study relates to the transformer's conspicuous attention. Subsequently, to suggest the network in this article, we combine the transformer with a convolutional network.

## 3. Dataset

The dataset utilized in this article, PlantDoc, published by researchers at the Indian Institute of Technology, is a collection of 13 plant species and 27 categories (including 17 types of diseases such as Bell Pepper Bacterial, Apple Black Rot, Cherry Powdery Mildew, Blueberry Healthy, Potato Early Blight, Corn Gray Spots, Grape Black Rot, and 10 types of healthy plants). The dataset contains 2,567 images, including 2,328 images in the training set and 239 images in the test set. The resolution of each image in the dataset is 416 × 416.

### 3.1. Dataset Analysis

**Figure 1:**
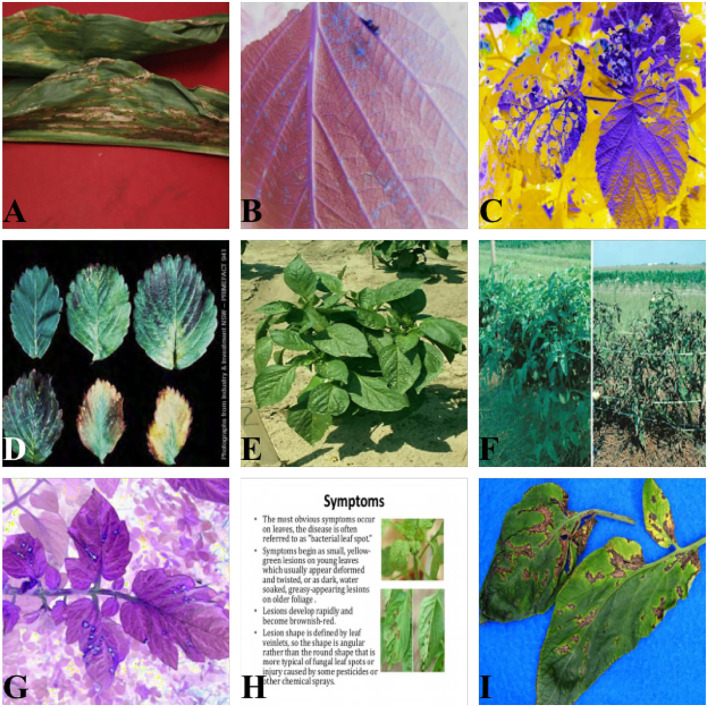
Dataset visualization. **(A,D,I)** Images on solid color backgrounds. **(B,C,G)** Images via color channel variation. **(E,F)** Images from practical production scenes. **(H)** Electronic document image.

**Figure 2:**
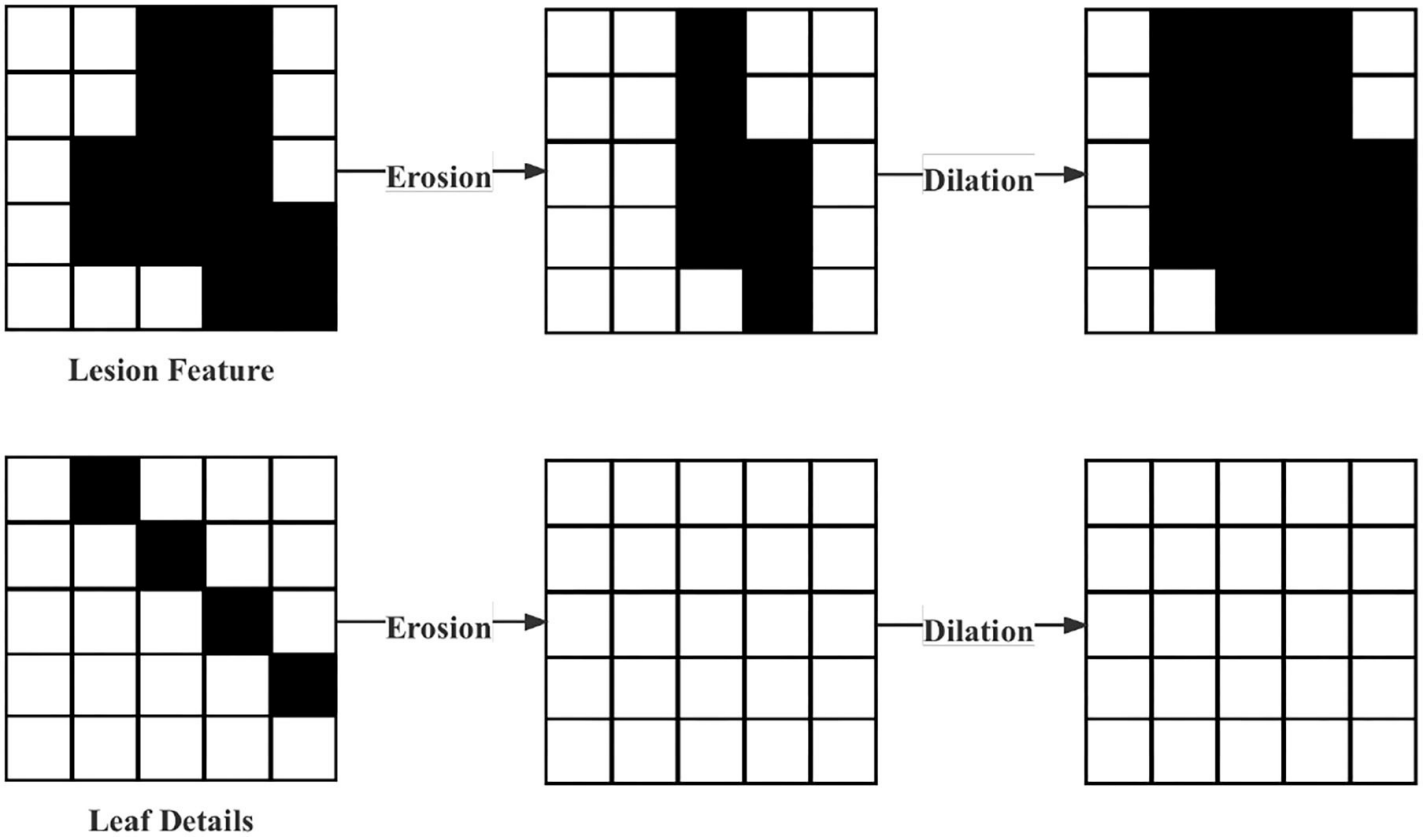
Processing of removal of interferential leaf details.

Figure 1 illustrates the following traits of the dataset adopted in this article:

1. This dataset contains a broad range of plants, and their diseased and healthy characteristics are more complex than traditional datasets. The leaves of one healthy plant may retain the visual characteristics of diseased leaves of another plant.
2. The source of the images in this dataset is complicated, including (1) images from practical production scenes, as illustrated in Figure 1E; (2) captured images on solid color backgrounds, as Figures 1A,I show; (3) and images that have undergone color channel variation as Figures 1C,G provided; (4) and even screenshots obtained from electronic documents, such as Figure 1H.
3. The scale of the images in this dataset varies greatly. There is only a partial image of one leaf in Figures 1B,D possesses six leaves, while Figures 1E,F comprise the whole plant with tens of leaves in total.

### 3.2. Data Augmentation

The above analysis reflects that although the dataset contains over 2,000 images, the multiple plant types and lesion types and the staggering differences in the images make it difficult to perform feature extraction. Nevertheless, deep learning models, particularly the CNN model, necessitate numerous data to undertake the training process. Therefore, it is necessary to undertake data augmentation on the dataset before performing feature extraction.

#### 3.2.1. Basic Augmentation

In this article, we referred to the method proposed by Krizhevsky et al. (2012), using image flipping, translation, and scaling for simple data augmentation. Image flipping and image translation improve the model's performance mainly by increasing the amount of data and enhancing the translation equivariance of the model. Due to image scaling, the model gains the ability to recognize different scales of targets, which promotes the robustness of the model. As mentioned in Section 3.1, the scales of the same targets are not the same, so it is crucial to improve the model's capacity to detect the same target at different scales. Image affine transformation is a specific implementation of image scaling.

The width and height of target images are anticipated to be *w*_*target*_ and *h*_*target*_, whereas those of the original image are *w*_*origin*_ and *h*_*origin*_. Formula (1) illustrates that when images are enlarged and shrunk, the Ω, which represents the scaling factor, is first defined. At that moment, we split the width and height of the original image through Ω. Afterward, we take a fragment inside the target frame, when the target frame's center point intersects with that of the processed image.


(1) $$\begin{array}{l} \Omega =min\left\{\frac{h_{target}}{h_{origin}},\frac{w_{target}}{w_{origin}}\right\} \end{array}$$

In parallel with the above-mentioned spatial and scale data enhancements, basic color channel transformations, such as HSV channel color variations, are applied in this article to enhance the recognition performance of the model under different lighting conditions.

#### 3.2.2. Advanced Augmentation

In addition to the basic data enhancement methods mentioned above, there are also some advanced augmentation methods to tackle the problem above.

Removal of interferential leaf details. Considering the characteristics of the dataset in this article, where many details in the leaf images will disturb the model. Therefore, erosion and dilation by Chen and Haralick (1995) were used in data pre-processing. First, the erosion operation is performed. The logical operation procedure is shown in Formula (2). Such an operation can not only remove the leaf details but also change the characteristics of the lesion, which is the reason why the following dilation process was necessary. Its logical operation process is shown in Formula (3). In Formula (2)–(3), A and B represent the original image of the operator, respectively. The expansion process can restore the original characteristics of the lesion. The operation process above is shown in Figure 2.


(2) $$\begin{array}{l} A⊙B=\left\{z|{\left(\hat{B}\right)}_{z}\subseteq A\right\} \end{array}$$

(3) $$\begin{array}{l} A⊕B=\left\{z|{\left(\hat{B}\right)}_{z}\cap A\neq \emptyset \right\} \end{array}$$

To address the huge memory loss and the network's suboptimal sensitivity to adversarial samples, we co-opted the Mixup method (Zhang et al., 2017). Mixup aims to solve the network's enormous memory loss and the lack of sensitivity to adversarial samples. Enhancing the sensitivity of the adversarial samples promotes the precision of the model because our model applied GAN modules. The method is shown in Formula (4)–(6).


(4) $$\begin{array}{l} \lambda =Beta\left(\alpha ,\beta \right) \end{array}$$

(5) $$\begin{array}{l} mixed\text{\_}batch_{x}=\lambda \times batch_{x1}+\left(1-\lambda \right)\times batch_{x2} \end{array}$$

(6) $$\begin{array}{l} mixed\text{\_}batch_{y}=\lambda \times batch_{y1}+\left(1-\lambda \right)\times batch_{y2} \end{array}$$

The Cutout (DeVries and Taylor, 2017) method stochastically removes partial samples and fills them with specific pixels while maintaining the classification result unaffected. The starting point of Cutout is identical to the random erasing method, aiming to enhance the generalizability and simulating masking. It arbitrarily picks a fix-sized square area and then utilizes all 0 fillings. However, the data should be subjected to a central normalization operation to avoid the influence of filling 0 values on training. Cutout enables CNNs to utilize the image's global information rather than local information consisting of small features.

Unlike Cutout, CutMix (Yun et al., 2019) remove parts of the sample and fill the removed parts with pixels from other samples. CutMix improves training efficiency by allowing the model to detect two objects from a local perspective of an image. It also helps the model concentrate on areas where distinguishing the objects is the toughest.

The Mosaic (Bochkovskiy et al., 2020) method can use more than one image simultaneously, which randomly uses 4 images, arbitrarily scaled, and then arbitrarily distributed for stitching, significantly augments the dataset, especially the random scaling adds multiple small objects, rendering the network more robust. At the same time, it enables the model to calculate the data of 4 images directly, renders expanding the mini-batch size unnecessary, and allows one GPU to get superior outcomes. The downside is that if the dataset itself has many small targets, then Mosaic data augmentation will cause the already small targets to become even smaller, resulting in poor generalization of the model.

**Figure 3:**
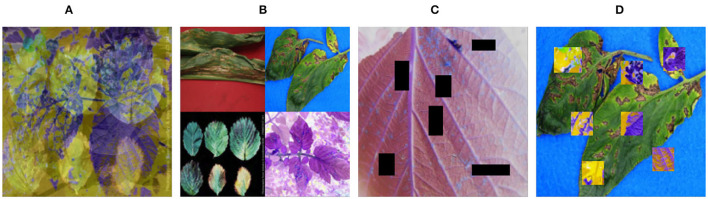
Demonstration of five date augmentation methods. **(A)** Mixup; **(B)** Mosaic; **(C)** CutMix; **(D)** CutOut.

Figure 3 displays these explicit effects.

## 4. Tranvolution Detection Network With GAN Modules

Mainstream one-stage object detection models—YOLO (Redmon et al., 2016; Redmon and Farhadi, 2017, 2018; Bochkovskiy et al., 2020) and SSD (Liu et al., 2016; Li and Zhou, 2017) are frequently utilized in target detection and have demonstrated outstanding performance on MS COCO (Lin et al., 2014) and Pascal VOC (Everingham, 2010) data sets. However, the characteristics of the YOLO series are not suitable for detecting leaf images.

As mentioned in the analysis of the dataset characteristics, there are high-density small object detection scenarios in practical applications. The general approaches to solving the small object detection problem include: increasing the resolution of the input image, which increases the computational complexity, and multi-scale feature representation, which makes the results uncontrollable. At present, the mainstream detection network incorporates the Feature Pyramid Network (FPN) (Lin et al., 2017). After the backbone extracts the features, the FPN contains the neck network with the fusion of deep feature maps and shallow feature maps. This structure improves the network's detection ability for different scales of objects. Nevertheless, it also complicates the network and has the possibility of overfitting. Therefore, this article proposes a multi-GANs structure, aiming to improve the above problem and enhance the model performance of the detection network. The main idea is to add a generative network model in front of the backbone of the detection network to augment the dataset. Subsequently, add a feature extractor based on the generative network model in the backbone of the detection network, improving CNN's feature extraction capability. The subsequent neck network and head network function will work more satisfactorily and efficiently when enough features are extracted.

Compared with the mainstream object detection models, including one-stage and two-stage, the main innovation of the Tranvolution detection network with GAN modules is:

1. Two generative network models are added to the network to address the inadequate training of CNNs due to small data sets and improve the ability of deep CNNs to extract image features.
2. We modified the ViT, by reducing the number of parameters, and improving the training speed, to improve CNN's ability to capture global features as a branch network. Because it inherits and combines the structural and global feature extraction advantages of CNN and visual transformers, the performance of the detection network is significantly better than CNN and vision transformers with comparable parameter complexity, showing the great potential capability in leaf image detection tasks.
3. Using label smoothing techniques and optimizing the loss function to improve the performance of the network.
4. Improve the NMS algorithm in the detection network by adding weight coefficients to fuse the bounding boxes.

**Figure 4:**
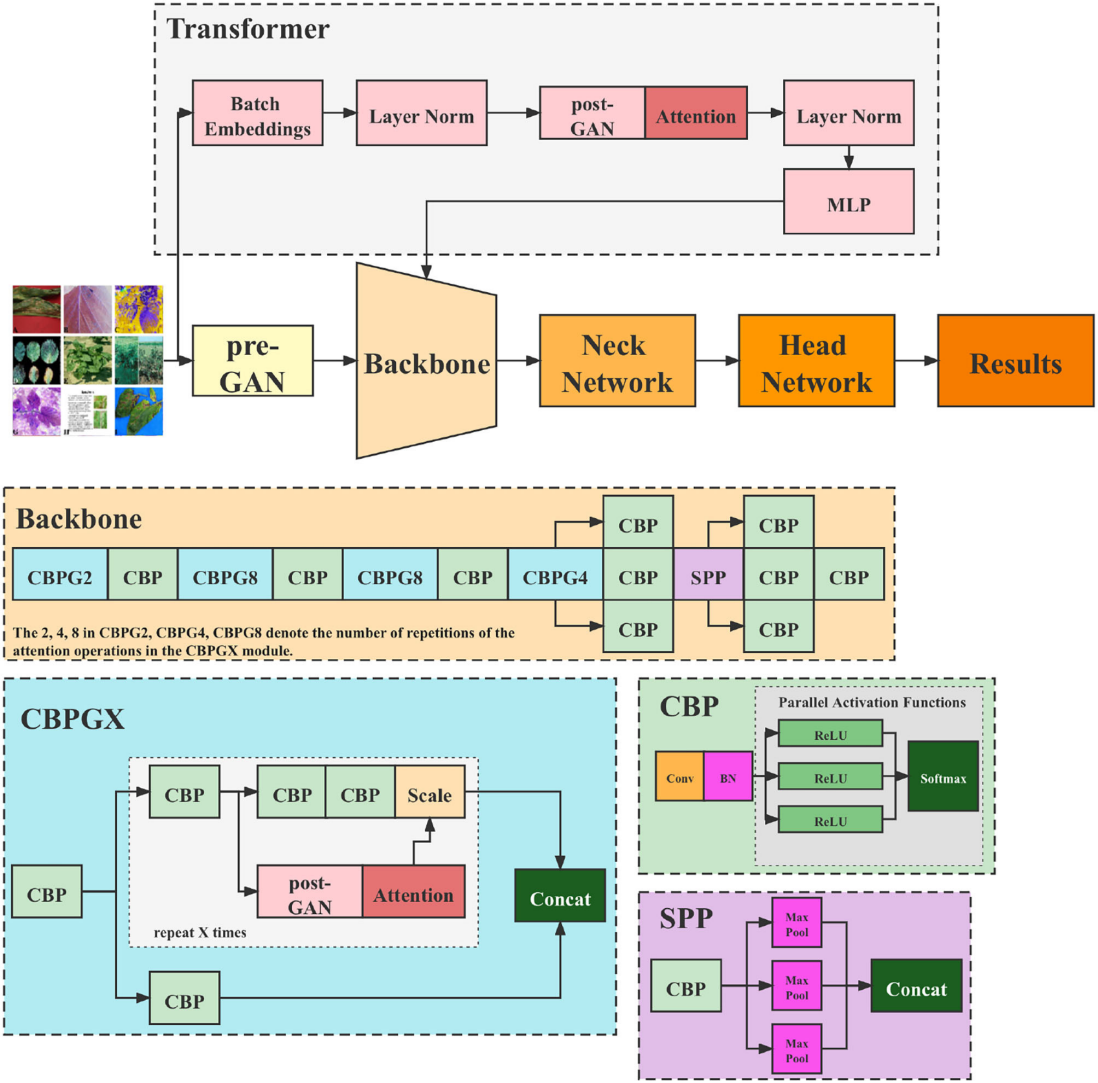
Structure of the Tranvolution detection network with GAN modules.

Figure 4 illustrates the structure of the Tranvolution detection network.

### 4.1. GAN Modules

Our network comprises two GAN modules: the pre-GAN and the post-GAN, as shown in Figure 4. Pre-GAN is placed before the backbone of the one-stage detection network to expand leaf images. The GAN module here can be implemented using various algorithms. Algorithm 1 shows the process of implementing pre-GAN using WGAN in the form of pseudo-code.

**Algorithm 1:** Algorithm of WGAN. α = 0.00005, *c* = 0.01, *m* = 64, *n*_*critic*_ = 5.

1: **Input**: dataset *D*
2: **Output**: dataset *D*′
3: **Require**: α, the learning rate. *c*, the clipping parameter. *m*, the batch size. *n*_*critic*_, the number of iterations of the critic per generator iteration.
4: **Require:** ω_0_, initial critic parameters. θ_0_, initial generator's parameters.
5: **while** θ has not converged **do**
6: **for** *t* = 0, ⋯, *n*_*critic*_ **do**
7: Sample ${\left\{x^{\left(i\right)}\right\}}_{i=1}^{m}\sim {\mathbb{P}}_{r}$ a batch from the real data.
8: Sample ${\left\{z^{\left(i\right)}\right\}}_{i=1}^{m}\sim p\left(z\right)$ a batch of prior samples.
9: $g_{w}\leftarrow {▽}_{\omega }\left\{\frac{1}{m}\overset{m}{\underset{i=1}{\sum }}f_{\omega }\left(x^{\left(i\right)}\right)-\frac{1}{m}\overset{m}{\underset{i=1}{\sum }}f_{\omega }\left(g_{\theta }\left(z^{\left(i\right)}\right)\right)\right\}$
10: ω←ω+α·*RMSProp*(ω, *g*_ω_)
11: ω←*clip*(ω, −*c, c*)
12: **end for**
13: Sample $\left\{{z^{\left(i\right)}}_{i=1}^{m}\sim p\left(z\right)\right\}$ a batch of prior samples.
14: $g_{\theta }\leftarrow {▽}_{\omega }\frac{1}{m}f_{\omega }\left(g_{\theta }\left(z^{\left(i\right)}\right)\right)$
15: θ←θ−α·*RMSProp*(θ, *g*_θ_)
16: **end while**

Another probable implementation of pre-GAN is the Balancing GAN (Mariani et al., 2018), introduced by IBM, which is a specific improvement on ACGAN (Odena et al., 2017), specifically designed to solve the problem of small sample size in unbalanced datasets.

**Figure 5:**
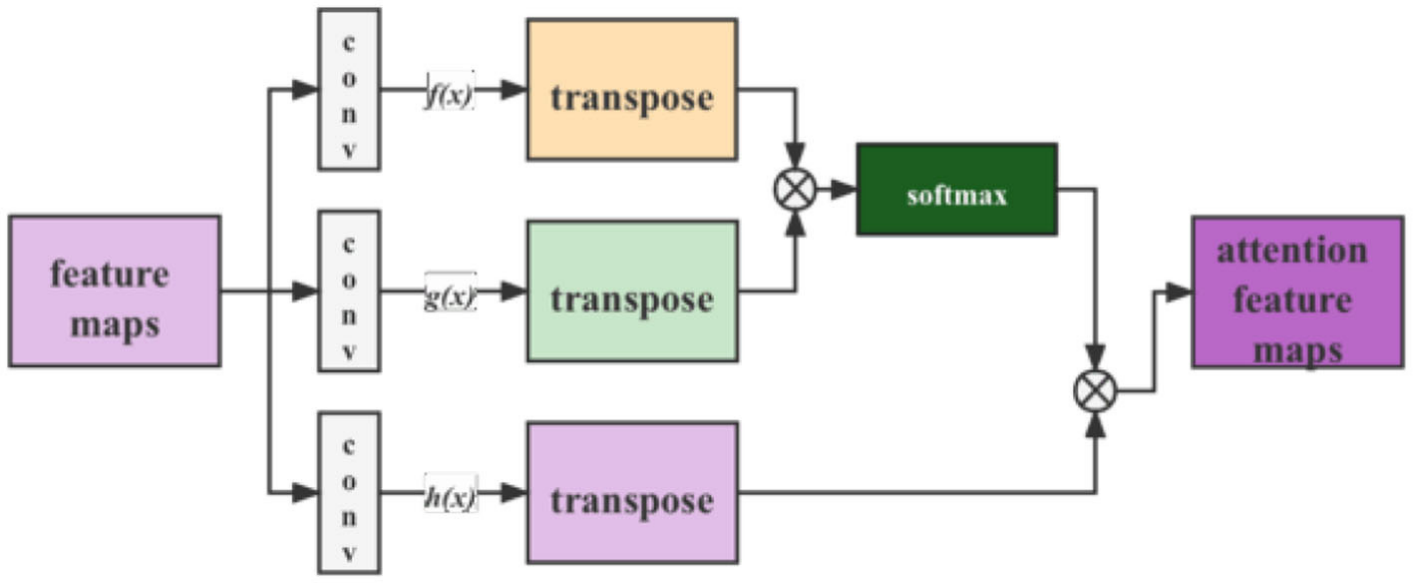
Flow chart of SAGAN.

The post-GAN locates in the attention mechanism module, as depicted in Figure 4. Its principal function is to add a noise mask to the feature maps taken from the backbone to enhance the model's robustness. The following Section 6 displays that introducing noise can considerably enhance model performance. The post-GAN module can be built in a variety of ways. SAGAN, e.g., is used as indicated in Figure 5.

These GAN modules enhance the detection network's robustness by adding GAN branches to regularize the results. In order to prevent the gradient from disappearing in the convolutional layer for inputs data more minor than zero, we changed the activation function for each block in the original model from ReLU to LeakyReLU. Meanwhile, e.g., the normalization layer works more satisfactorily on generative tasks than the batch normalization layer, the batch normalization layers of the GAN module were replaced with instance normalization layers.

It should be noted that the GAN module in the network is pre-trained and does not participate in the training of the monitoring network. In fact, since the significance of the GAN in this article is to add noise to the image, we do not expect the noise patterns to affect object detection. Therefore, a good criterion for noise addition is that it can bring appropriate occlusion, blurring, or interference to the original image but not destroy the object features. Therefore, we used the original image as the input and the artificially enhanced image as the training set to train the GAN. For determining whether the GAN model has completed training, we referred to the most common metric, the inception score. In addition, we undertook combination and comparison experiments to select trained GAN models and the combination strategy.

### 4.2. Tranvolution

In 2020, the transformer achieved extraordinary classification, detection, and segmentation results. However, the drawbacks are apparent:

1. Its training time is exceedingly long;
2. It is not conducive to deployment acceleration;
3. It requires a vast dataset;

Therefore, in this article, we referred to the idea of a transformer and design it as a branch network, which exploits its ability to extract global features and relies on the CNN backbone, avoiding its training time from being too prolonged.

As Figure 4 depicts, CNN backbones still utilize the feature pyramid structure. Moreover, the feature map's resolution decreases as the depth of the network increases while the number of channels increases. The transformer branch network is responsible for providing global features to the backbone. First, the input image is divided into patches, and then mainly undertake transformation for each patch as a flattening operation. For instance, assuming the input image size is 256 × 256, if it is divided into 64 patches, each is 32 × 32 in size. The original Transformer encoder is composed of alternating multi-heads self-attention and multi-layer perceptron. Nevertheless, in this article, to reduce the number of parameters and training time, this part is transformed into the identical mechanism as the attention module in the backbone, i.e., attention based on post-GAN optimization. After being processed through the layer norm layer, all the features are pooled and sent to the CNN backbone.

### 4.3. Loss Function

The detection network's loss function is composed of three portions: regression box loss, *CIoU* loss, and classification loss. The calculation process is shown in Formula (7)–(10). Box coordinate error (*x*_*i*_, *y*_*i*_) denotes the predicted box's center position coordinate, and (*w*_*i*_, *h*_*i*_) is its width and height. $\left(\hat{x_{i}},\hat{y_{i}}\right)$ and $\left(\hat{w_{i}},\hat{h_{i}}\right)$ denote coordinates and size of the labeled ground truth box, respectively. Furthermore, λ_*coord*_ and λ_*noobj*_ are constants. *K* × *K* represents the grids' amount. *M* expounds the predicted boxes' overall amount. Besides, $I_{ij}^{obj}$ is one when the *ith* grid detects a target and zero otherwise.


(7) $$\begin{array}{l} Loss=Loss_{bounding\text{\_}box}+Loss_{ciou}+Loss_{classification} \end{array}$$

(8) $$\begin{array}{l} Loss_{bounding\text{\_}box}=\lambda_{coord}\overset{K\times K}{\underset{i=0}{\sum }}\overset{M}{\underset{j=0}{\sum }}I_{ij}^{obj}\left(2-w_{i}\times h_{i}\right)\left[{\left(x_{i}-{\hat{x}}_{i}\right)}^{2}+{\left(y_{i}-{ŷ}_{i}\right)}^{2}\right]+\\ {\;\lambda }_{coord}\overset{K\times K}{\underset{i=0}{\sum }}\overset{M}{\underset{j=0}{\sum }}I_{ij}^{obj}\left(2-w_{i}\times h_{i}\right)\left[{\left(w_{i}-{ŵ}_{i}\right)}^{2}+{\left(h_{i}-{ĥ}_{i}\right)}^{2}\right] \end{array}$$

(9) $$\begin{array}{l} Loss_{ciou}=\overset{K\times K}{\underset{i=0}{\sum }}\overset{M}{\underset{j=0}{\sum }}I_{ij}^{obj}\left[{Ĉ}_{i}log\left(C_{i}\right)+\left(1-{Ĉ}_{i}log\left(1-C_{i}\right)\right)\right]+\\ {\;\lambda }_{noobj}\overset{K\times K}{\underset{i=0}{\sum }}\overset{M}{\underset{j=0}{\sum }}I_{ij}^{noobj}\left[{Ĉ}_{i}log\left(C_{i}\right)+\left(1-{Ĉ}_{i}log\left(1-C_{i}\right)\right)\right] \end{array}$$

(10) $$\begin{array}{l} Loss_{classification}=\sum_{i=0}^{K\times K}I_{ij}^{obj}\sum_{c\in classes}\left[{\hat{p}}_{i}(c)log(p_{i}(c)\right)\\ \;+(1-{\hat{p}}_{i}(c)log(1-p_{i}(c))] \end{array}$$

### 4.4. Fusion Method for Bounding Boxes

We proposed a new fusion algorithm for bounding boxes that gave up the NMS solution of removing bounding boxes with low confidence and adopted the method of fusing different bounding boxes of the same object. In fact, the weight coefficients *s* were introduced in the fusion process, and the weight coefficients of each bounding box were calculated as shown in Formula (11).


(11) $$\begin{array}{l} C_{s}=\alpha \times A_{s}+\left(1-\alpha \right)\times B_{s} \end{array}$$

The *alpha* represents the sub-network weights of the generative sub-network, and by adjusting the size of α, we can control the degree of influence that the generative sub-network on the main detection network; (*x*_1_, *y*_1_), (*x*_2_, *y*_2_) represent the top-left and bottom-right coordinates of a box, respectively; c represents the confidence level of a bounding box. So the higher the *c* box is, the larger *s* is, and it contributes more to the process of generating a new box. The shape and position of the new box are closer to boxes with larger weights.


(12) $$\begin{array}{l} C_{x_{1}}=\frac{A_{x_{1}}\times A_{s}+B_{x_{1}}\times B_{s}}{A_{s}+B_{s}} \end{array}$$

(13) $$\begin{array}{l} C_{y_{1}}=\frac{A_{y_{1}}\times A_{s}+B_{y_{1}}\times B_{s}}{A_{s}+B_{s}} \end{array}$$

(14) $$\begin{array}{l} C_{x_{2}}=\frac{A_{x_{2}}\times A_{s}+B_{x_{2}}\times B_{s}}{A_{s}+B_{s}} \end{array}$$

(15) $$\begin{array}{l} C_{y_{2}}=\frac{A_{y_{2}}\times A_{s}+B_{y_{2}}\times B_{s}}{A_{s}+B_{s}} \end{array}$$

Formula (12)–(15) shows how to get the fused bounding box C by using two bounding boxes A and B.

## 5. Experiment

### 5.1. Evaluation Metrics

This study uses five metrics to validate the performance of our model, including Precision (*P*), Recall (*R*), *mAP, IoU*, and FPS.

Precision (*P*) denotes the proportion of the number of actual positive samples in the correct prediction sample (True Positive, *TP*) to the number of all positive samples (True Positive, *TP* and False Positive, *FP*). Precision is calculated as:


(16) $$\begin{array}{l} P=\frac{TP}{TP+FP} \end{array}$$

Recall (*R*) denotes the proportion of the number of actual positive samples in the correctly predicted sample (True Positive, *TP*) to the number of actual positive samples (True Positive, *TP* and False Negative, *FN*). The Recall is calculated as:


(17) $$\begin{array}{l} R=\frac{TP}{TP+FN} \end{array}$$

Intersection over Union (*IoU*) is a criterion for measuring the accuracy of detecting corresponding objects in a given dataset. *IoU* can be used for any task that yields a prediction range (bounding boxes) in the output. Given a set of images, *IoU* presents the similarity between the predicted and ground-truth regions of the objects present in the image and is defined by the following equation:


(18) $$\begin{array}{l} IoU=\frac{\left|A\cap B\right|}{\left|A\cup B\right|}=\frac{TP}{TP+FP+FN} \end{array}$$

where *A* presents the ground truth region and *B* expounds the predicted part. The value of *IoU* ranges in the interval 0 to 1 (inclusively). More specifically, 0 means that the predicted region and the ground-truth region have no overlap; 1 means that the predicted region and the ground-truth region overlap entirely. Meanwhile, when the value of *IoU* ≥ 0.5, this situation is regarded as a true positive; otherwise, it is a false positive situation.

Neither Precision nor Recall alone is a good criterion of a model's performance, and in reality, generally, if Accuracy is high, Recall will be relatively low, and vice versa. So finding a balance between Precision and Recall is significant. One way is to draw a Precision-Recall curve (PR curve) with Recall on the horizontal axis and Precision on the vertical axis. The area under the Precision-Recall curve is used to measure, and the value is called Average Precision (*AP*).

In practice, the *IoU* of an image's predicted and ground-truth regions is calculated foremost. The outcome can be concluded to be *TP* or *FP* in terms of the threshold of the *IoU*. Subsequently, sort the confidence of each predicted region from high to low and then attain Precision and Recall under different confidence thresholds. According to these sets of Precision and Recall values, draw the corresponding PR curve and calculate the *AP* value.

Average Precision measures the effectiveness of one detection category, and mean Average Precision (*mAP*) assesses the detection effectiveness of multiple categories. The *mAP* is obtained by averaging the AP values of all classes.

The number of Frames Per Second, also referred to as *FPS*, is a measure of the amount of information used to save and display dynamic video. The more frames per second, the smoother the action displayed will be. In a deep learning model for object detection, *FPS* is used to represent the inference speed of the model.

### 5.2. Experiment Setting

A personal computer (CPU: Intel(R) i9-10900KF; GPU: NVIDIA RTX 3080 10 GB; Memory: 16 GB; OS: Ubuntu 18.04, 64 bits) is used to carry out the entire model training and validation process. The Adam optimizer with an initial learning rate, *a*_0_ = 1*e*^−4^ is selected in this article, and the learning rate increment is adjusted using the method specified in Section 5.4.

### 5.3. Label Smoothing

Usually, there are a small number of mislabels in machine learning samples, which can affect the prediction effect, especially when the sample size is small. Therefore, in this article, we adopted the label smoothing technique to improve the situation, which is based on the following solution: to avoid “over-trusting” the labels of training samples by assuming that some of the labels may be incorrect at the time of training.

At each iteration, instead of inputting (*x*_*i*_, *y*_*i*_) directly into the training set, an error rate ϵ is set, and (*x*_*i*_, *y*_*i*_) is substituted into the training with probability 1 − ϵ, and (*x*_*i*_, 1 − *y*_*i*_) is substituted into the training with probability ϵ. In this way, the model is trained with both correct and incorrect label inputs, and it is conceivable that the model so trained will not match every label “to the fullest extent” but only to a certain extent. This way, the model will be less affected if there are indeed incorrect labels.

When we use cross-entropy to describe the loss function, for each sample *i*, the loss function is:


(19) $$\begin{array}{l} Loss_{i}=-y_{i}\times P\left(\hat{y_{i}}=1|x_{i}\right)-\left(1-y_{i}\right)\times P\left(\hat{y_{i}}=0|x_{i}\right) \end{array}$$

After randomization, the new labels have the same probability of 1 − ϵ as *y*_*i*_ and a different probability of ϵ, i.e., 1 − *y*_*i*_. Therefore, when the randomized labels are used as training data, the loss function has the same probability of 1 − ϵ as the above equation and the probability of ϵ as:


(20) $$\begin{array}{l} Loss_{i}=-\left(1-y_{i}\right)\times P\left(\hat{y_{i}}=1|x_{i}\right)-y_{i}\times P\left(\hat{y_{i}}=0|x_{i}\right) \end{array}$$

After weighted averaging Formula (19) and (20) by probability, having $y_{i}^{\prime }=\epsilon \times \left(1-y_{i}\right)+\left(1-\epsilon \right)\times y_{i}$, we can obtain:


(21) $$\begin{array}{l} Loss_{i}=-\left(1-y_{i}^{\prime }\right)\times P\left(\hat{y_{i}}=1|x_{i}\right)-y_{i}^{\prime }\times P\left(\hat{y_{i}}=0|x_{i}\right) \end{array}$$

Compared with the original cross-entropy expression, only *y*_*i*_ is replaced with $y_{i}^{\prime }$, while everything else remains the same. This is actually equivalent to replacing each label *y*_*i*_ with $y_{i}^{\prime }$ and then performing the regular training process. Therefore, in this article, randomization was not conducted before training except by replacing each label accordingly.

### 5.4. Training Strategy

Warm-up (He et al., 2016) is a training strategy. The *exp* warm-up method is examined in this article, which involves linearly accelerating the learning from a minuscule value to the predefined learning speed and then fading in terms of the *exp* function law. This article also tried *cos* Warm-up. According to the *cos* function law, the learning rate increased linearly from a minimal value to a preset value and then decayed. The principle of cosine decay is shown in Formula (22).


(22) $$\begin{array}{l} \eta_{t}=\eta_{min}^{i}+\frac{1}{2}\left(\eta_{max}^{i}-\eta_{min}^{i}\right)\left(1+cos\left(\frac{T_{cur}}{T_{i}}\pi \right)\right) \end{array}$$

Among it, *i* represents the number of iterations, $\eta_{max}^{i}$ and $\eta_{min}^{i}$ represent the maximum and minimum values of the learning rate, respectively, *T*_*cur*_ represents the number of epochs currently executed. In contrast, *T*_*i*_ represents the overall number of epochs in the number *i* step.

## 6. Results

In this section, the model introduced in Section 4 was implemented for object detection in leaf images. We trained the datasets with three input sizes, 300 × 300, 416 × 416, and 608 × 608, which are the suggested input resolutions for the YOLO model.

### 6.1. Validation Results

In this section, a comparison of various models and different pre-training parameters is provided, where MolileNet and Faster-RCNN's data are acquired from PlantDoc (Singh et al., 2020). Table 1 illustrates the statistical results. The best results of the index are marked red.

**Table 1:** Comparisons of different detection networks' performance (in %).

**Model**	**Input size**	**Pretrained weights**	**Precision**	**Recall**	**mAP (at 50% IoU)**	**FPS**
MobileNet	416 × 416	COCO	-	-	32.8 Singh et al., 2020	-
MobileNet	416 × 416	COCO + PVD	-	-	22.4 Singh et al., 2020	-
Faster-RCNN-Inception-ResNet	416 × 416	iNaturalist	-	-	36.1 Singh et al., 2020	-
Faster-RCNN-Inception-ResNet	416 × 416	COCO	-	-	38.9 Singh et al., 2020	-
SSD	300 × 300	COCO	37.9	39.4	38.3	44
FSSD	300 × 300	COCO	39.7	36.3	37.6	39
RefineDet	300 × 300	COCO	34.4	38.3	35.9	43
EfficientDet	416 × 416	COCO	42.1	39.2	39.7	35
YOLO v3	608 × 608	COCO	39.7	39.4	39.5	88
YOLO v4	608 × 608	COCO	41.4	39.5	38.1	87
YOLO v5	608 × 608	COCO	45.0	38.6	41.7	97
Ours	416 × 416	COCO	51.7	48.1	50.3	37

*The red values annotate the evaluation metrics of our model and represent they are the best among all of the models*.

As demonstrated in Table 1, YOLO v5 (Jocher, 2020) has the best speed, with *FPS* reaching 97, although it possesses the highest resolution. The *mAP* of MobileNet, the pre-training parameters fetched by adopting COCO and PVD, is 22.4%, which is the worst performance of all models. These *P*, *R*, and *mAP* of YOLO v5 are the most excellent among all YOLO series, reaching 45.0, 38.6, and 41.7% to be exact. Meanwhile, this performance exceeds any other comparable models. The most similar model to YOLO v5 is EfficientDet, with *P*, *R*, and *mAP* of 42.1, 39.2, and 39.7%, respectively. Nevertheless, its inference speed is significantly lower than YOLO v5, reaching only 36% of the latter. That is probably due to the stronger performance of the attention extraction module in YOLO v5. SSD series' performance, on the whole, lags behind the YOLO series and EfficientDet, and the YOLO series are the best models among the comparisons. For the backbone part of the proposed model, we selected the pre-training parameters obtained based on ImageNet. Moreover, its *Precision*, *Recall*, and *mAP* are superior to other comparable models. However, our model fails to be superior in inference speed (only 38% of YOLO v5). The complexity of the GAN modules and Transformer branch cause it. As depicted above, the Tranvolution detection network with GAN modules reflects the best detection performance on the validation set, according to the results.

### 6.2. Detection Results

**Figure 6:**
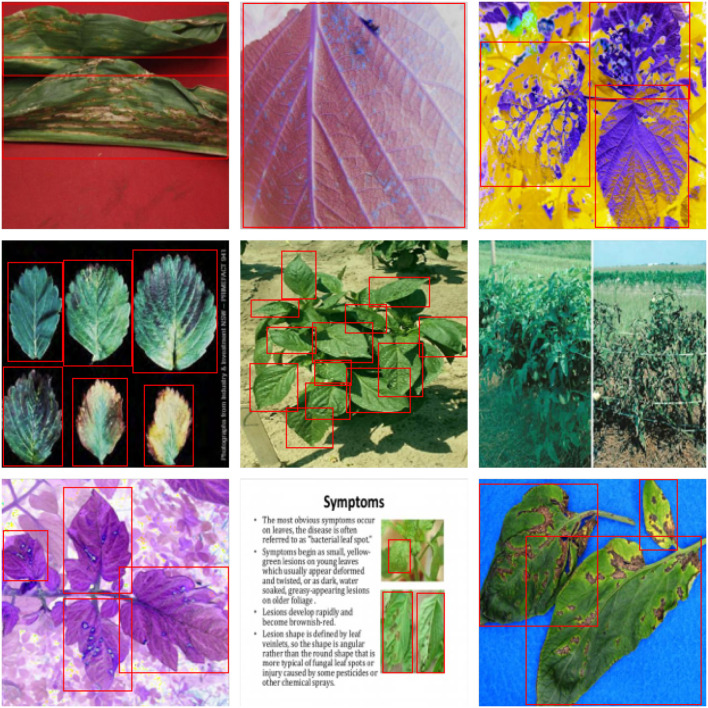
The ground truth in the dataset.

**Figure 7:**
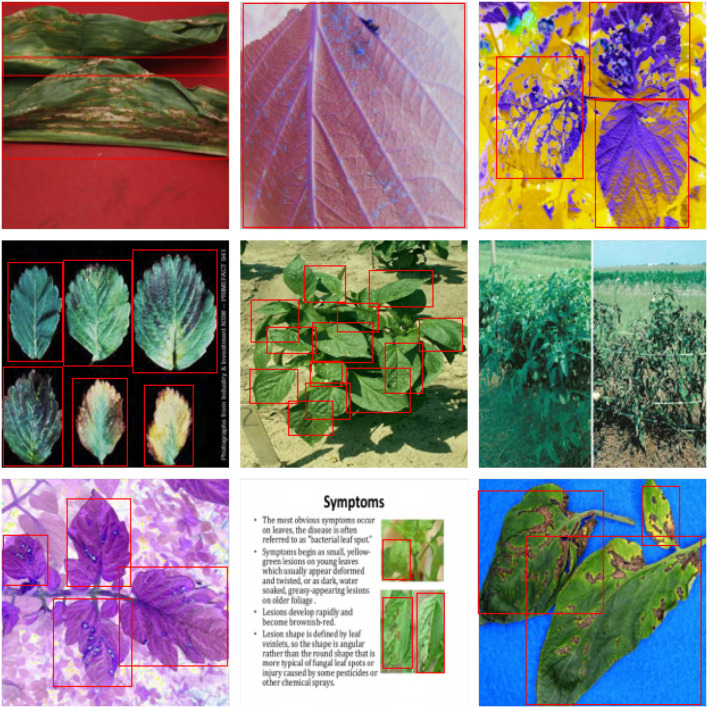
The detection results of YOLO v3 in the dataset.

**Figure 8:**
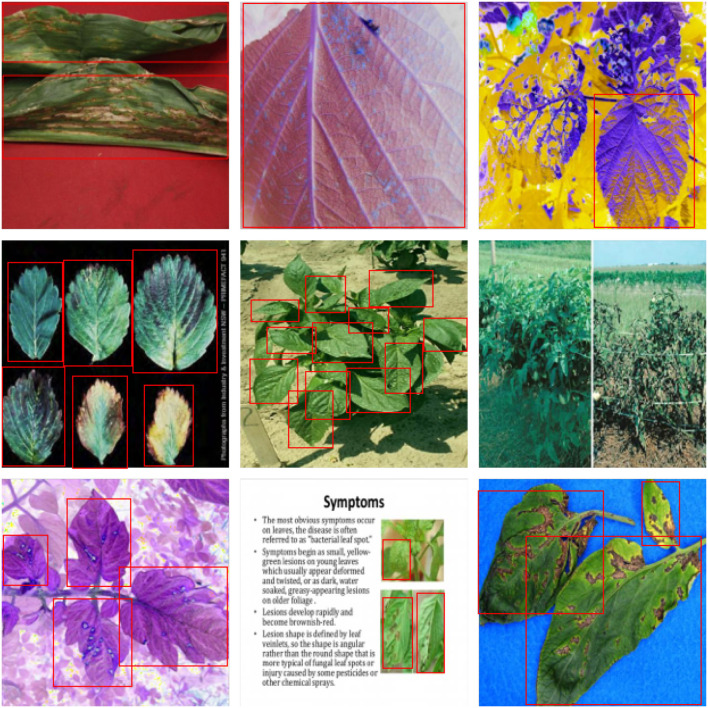
The detection results of SSD in the dataset.

**Figure 9:**
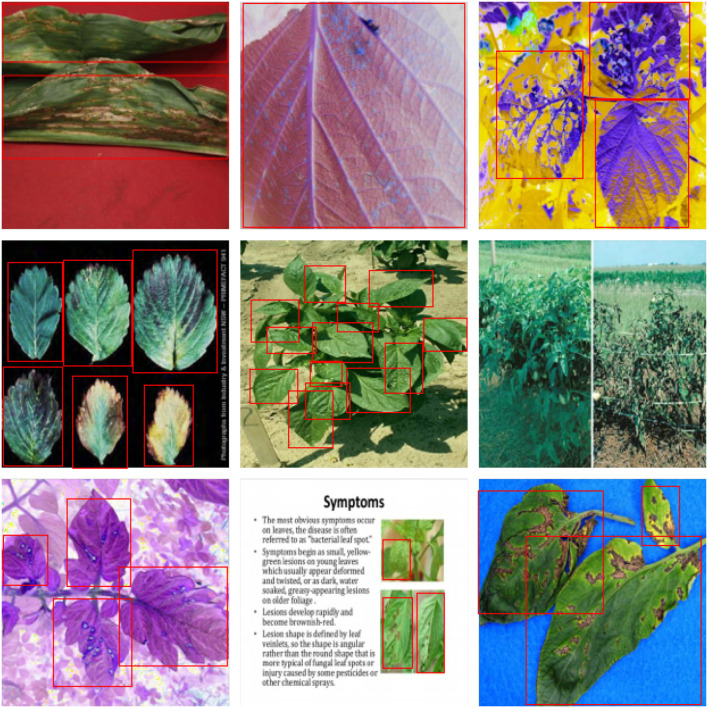
The detection results of EfficientDet L2 in the dataset.

**Figure 10:**
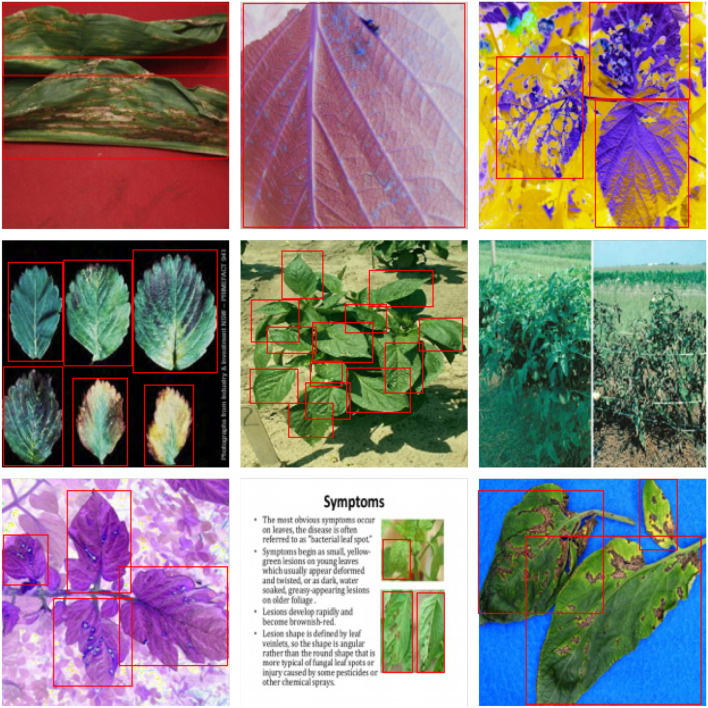
The detection results of our model in the dataset.

For further comparison, we extracted nine images from the test set, which is identical to the nine images shown in Figure 1. The reason for using these nine images for this presentation is that these images show as many detection scenarios as possible in the dataset. Figures 6–10 depicts the detection results. Figure 6 denotes the ground truth; the red boxes in the rest of the images denote the predicted bounding boxes.

It can be witnessed that the SSD series performs very poorly in these nine images, while EfficientDet and YOLO series perform relatively well and detect lesions accurately. However, when the detected objects are too tiny, all models' performance decreases, and part of the models even have some unlabeled detected objects. This situation is probably related to the attention extraction module in these networks. Our model outperforms the previous models by highly accurate object detection, even when detecting moderately dense objects. Although there is still room for improvement, it has outperformed other models. On the one hand, we augment the image with the WGAN model before it is fed into the backbone. On the other hand, we add the SAGAN model to the attention extraction module, which can significantly improve the model's robustness.

## 7. Discussion

### 7.1. Ablation Experiment of GAN Modules

This article uses GAN modules in backbone and attention extraction modules, while GAN models have many branches and focus. The primary purpose of the pre-GAN module in front of the backbone is to enhance the model input. In contrast, the post-GAN module in the attention extraction module generates an attention mask to enhance the model's robustness. Therefore, for the two GAN modules with different purposes, different GAN models are implemented in this article, including WGAN, BAGAN, SAGAN, and SPA-GAN. Several ablation experiments are conducted, and the experimental results are shown in Table 2.

**Table 2:** Results of different implements of GAN modules (in %).

**Method**	**Precision**	**Recall**	**mAP**	**FPS**
No GAN (baseline)	39.3	37.8	38.5	63
WGAN + SAGAN	51.7	48.1	50.3	37
BAGAN + SAGAN	49.8	49.1	49.3	37
WGAN + SPA-GAN	51.9	47.6	49.7	37
BAGAN + SPA-GAN	48.1	46.3	46.6	37

Table 2 reflects that using WGAN and SAGAN to implement pre-GAN and post-GAN, respectively, can optimize the model performance, with the three primary metrics reaching 51.7, 48.1, and 50.3%. As a comparison, WGAN is better than BAGAN in the choice of pre-GAN. Regardless of the implementation of the post-GAN, this is probably because BAGAN uses a different formula from WGAN in computing the difference between the generated data and the original data, failing to maximize the generator's and discriminator's performances. In addition, as revealed in the experiments, the inference speed of the model is almost the same regardless of the combination type of GAN modules used. More precisely, the inference speed is only 10 ms slower compared to the baseline. However, it is apparent that the GAN modules can significantly improve the model's performance regardless of the implementation approaches. Therefore, we argue that although the addition of GAN modules slows down the model, the optimization is reasonable considering performance improvement.

**Figure 11:**
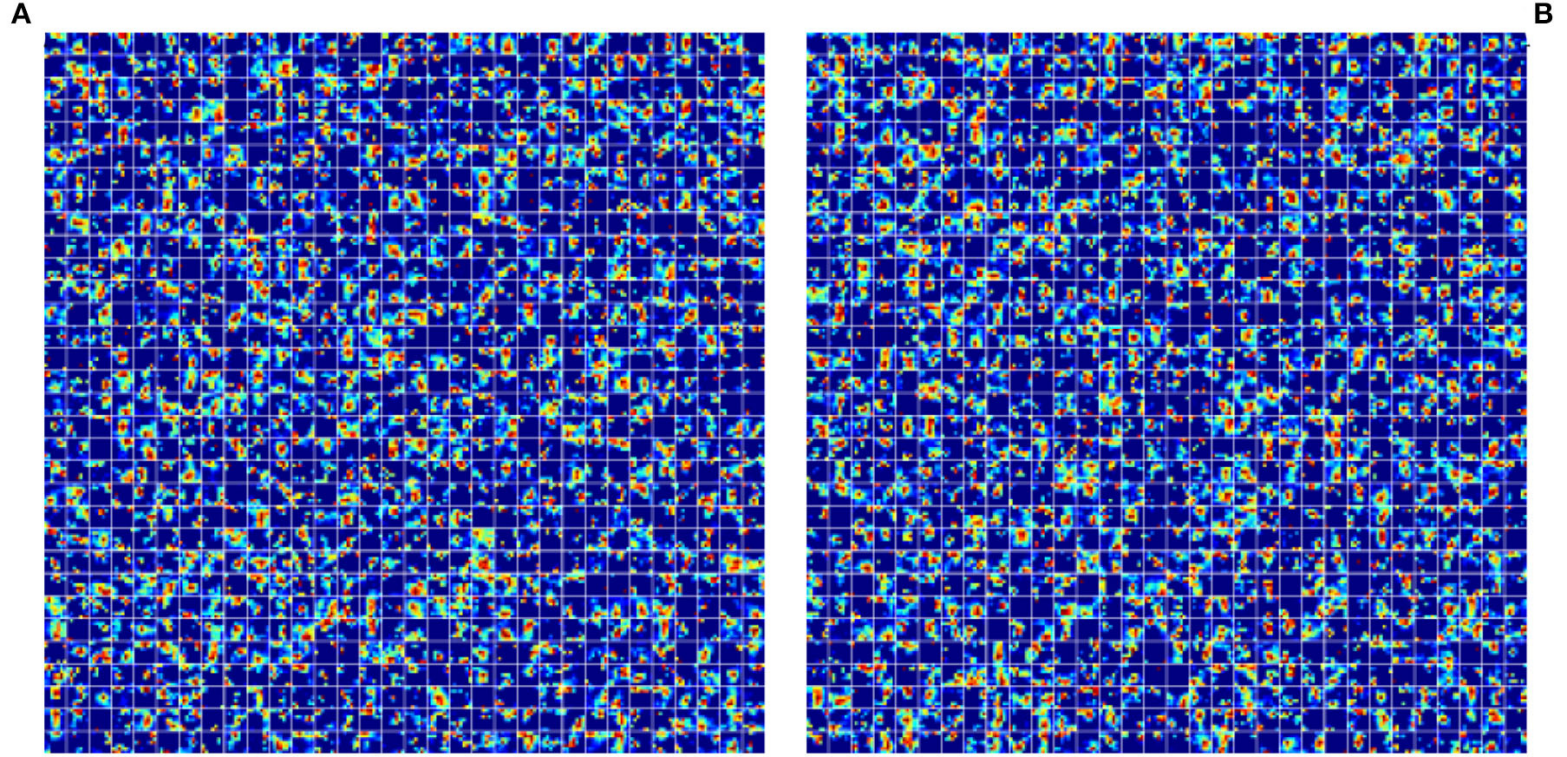
Illustration of noise mask generated by different GAN models. **(A)** Feature maps generated by WGAN. **(B)** Feature maps generated by SAGAN.

Moreover, Self-Attention Generative Adversarial Networks (SAGAN) contain an attention mechanism, and the transformer's structure is also for feature extraction; both network models have the same effect on the model in this respect. As shown in the table above, the model with the combination of WGAN + SAGAN has the best performance, which is probably related to the self-attention mechanism in SAGAN. The self-attention mechanism has been widely used in the field of machine translation, and the transformer was first used in the field of natural language processing. The incorporation of transformer and SAGAN with self-attention also achieved the best performance in the object detection task in this article, which made us quite excited. We will further explore how these two mechanisms work specifically in computer vision later. We tried to visualize the mask of noise generated by two post-GANs, as shown in Figure 11.

As Figure 11 depicts, although the feature maps generated by the two post-GAN implementing approaches differ dramatically, the resulting highlights are essentially the same. It is difficult to comprehend in the traditional human style of thinking and reading as noise mask affects feature maps. Yet, the noise generation area is nearly identical because the noise mask can substantially increase the model's performance. We hypothesize that the post-GAN module can improve the model's resilience by adding noise to the object area.

### 7.2. Ablation Experiment of Pre-processing Methods

To verify the effectiveness of the various pre-processing methods proposed in Section 3.2.2. The ablation experiments were performed on our model. The experimental results are shown in Table 3.

**Table 3:** The ablation experiment results from different pre-processing methods (in %).

**MixUp**	**CutOut**	**CutMix**	**Mosaic**	**Precision**	**Recall**	**mAP**
✓	✓	✓	✓	51.7	48.1	50.3
✓	✓	✓		51.2	48.9	50.5
✓	✓		✓	50.4	48.3	49.8
✓		✓	✓	50.4	48.4	49.8
	✓	✓	✓	51.7	48.2	50.3

Table 3 indicates that the CutOut method and CutMix method are the most effective for data enhancement. In contrast, the CutMix method does not appear to have a more positive effect than the above combinations, because the model works best merely when three data enhancement methods—the CutOut, CutMix, and Mosaic method—are used.

### 7.3. Hardware Deployment Application

To deploy the model proposed in this article into a practical application scenario, the model is packaged and deployed in conjunction with the Hbrid E203 RISC-V processor. The main reason for choosing this hardware is that it depends on an open-source RISC-V platform and can be highly customized. However, considering its computational power, there is still a need to optimize the computational process of the model in this article. In this article, we borrowed Strassen's (Strassen, 1969) optimization idea to optimize the matrix multiplication because the convolutional layer in CNN uses a lot of matrix multiplication operations, so optimizing the efficiency of matrix multiplication operations can significantly improve the model inference speed. This scheme has the following contributions:

1. We encapsulate the model proposed in this article and save the parameters of the trained model so that the inference process runs locally;
2. We use Strassen's algorithm to optimize the matrix multiplication method;
3. We developed on the Hbird E203 platform, and the model hardware is deployed.

As shown in Algorithm 2, the time complexity of the convolution operation using the conventional matrix multiplication is θ(*n*^3^), where *n* is the matrix dimension.

**Algorithm 2:** Matrix multiplication algorithm.

1: **input:** matrix A, matrix B
2: **output:** matrix C
3: n = A.rows
4: create a new *n* × *n* matrix C
5: **for** *i* = 1 **to** *n*
6: **for** *j* = 1 **to** *n*
7: *C*_*ij*_ = 0
8: **for** *k* = 1 **to** *n*
9: *C*_*ij*_ = *C*_*ij*_ + *A*_*ik*_ · *B*_*kj*_
10: **return** matrix C

To accelerate the inference speed of the model, we used the Strassen algorithm, which is essentially a partitioning method with time complexity $\theta \left(n^{log_{2}^{7}}\right)$, to optimize the matrix multiplication operation. The procedure is shown in the Algorithm 3.

**Algorithm 3:** Strassen algorithm.

1: **input:** matrix A, matrix B
2: **output:** matrix C
3: n = A.rows
4: create a new *n* × *n* matrix C
5: **if** *n* == 1
6: *C*_11_ = *A*_11_ · *B*_11_
7: **else**
8: devide matrix A into r sub-matrices *A*_11_, *A*_12_, *A*_21_, *A*_22_
9: devide matrix B into r sub-matrices *B*_11_, *B*_12_, *B*_21_, *B*_22_
10: devide matrix C into r sub-matrices *C*_11_, *C*_12_, *C*_21_, *C*_22_
11: *C*_11_ = *Strassen*(*A*_11_, *B*_11_)+*Strassen*(*A*_12_, *B*_21_)
12: *C*_12_ = *Strassen*(*A*_11_, *B*_12_)+*Strassen*(*A*_12_, *B*_22_)
13: *C*_21_ = *Strassen*(*A*_21_, *B*_11_)+*Strassen*(*A*_22_, *B*_21_)
14: *C*_22_ = *Strassen*(*A*_21_, *B*_12_)+*Strassen*(*A*_22_, *B*_22_)
15: **return** matrix C

**Figure 12:**
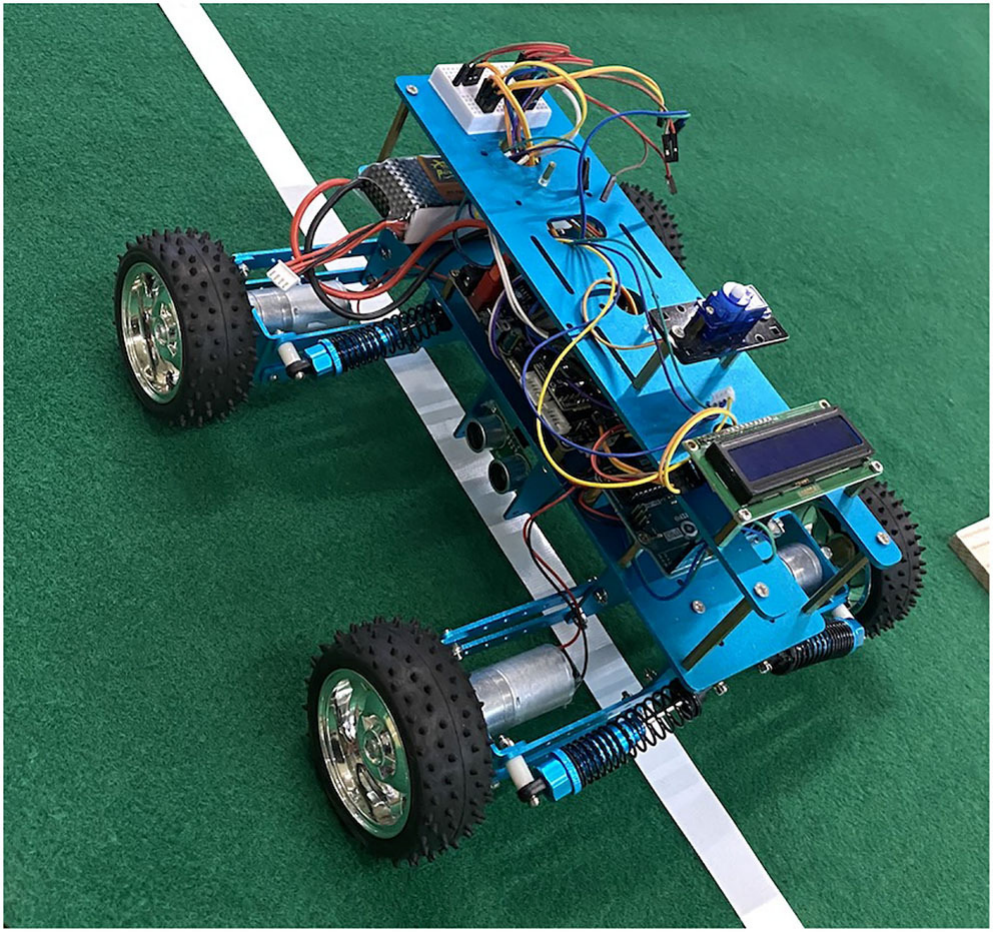
Intelligent agricultural robot, with infrared distance measurement and multiple cameras deployed on the bottom.

Figure 12 displays the intelligent agricultural robot based on the above chip and algorithm, which can realize the self-tracing function and leaf disease detection through the infrared distance measurement and camera on the bottom of the vehicle body.

### 7.4. Limitation

Although the model proposed in this article has surpassed other comparative models in both evaluation metrics and detection results, it still has limitations. As shown in Figure 10, our model's defects are pronounced in the task of small-scale object detection. Therefore, we analyzed the model performance on different subclasses of the dataset, as shown in Table 4.

**Table 4:** Comparisons of our model performance on different leaf size sub-dataset (in %).

**Leaf size**	**Precision**	**Recall**	**mAP (at 50% IoU)**
Small	38.9	32.7	47.8
Medium	73.8	67.5	70.6
Large	95.1	88.3	89.4

Table 4 reflects that our model does not perform well when there are too many objects, and there is some mislabeling. However, this result is perfectly acceptable in practical application scenarios when comparing and referring to specific images. Moreover, considering that the dataset is annotated from the web, some leaves are not annotated manually, but our model annotates them. Although this is only a minimal number of mislabeling cases, it can be seen that our model cares more about whether all of them are detected, so some of them are mislabeled. In the plant protection scenario, we think the loss caused by mislabeling is much lower than that caused by detection missing. In summary, we will further improve the performance of our model in object-intensive scenarios in the future.

## 8. Conclusion

Considering agriculture's irreplaceable significance in human social development, and with the cutting-edge technology's progress in object detection, plant disease detection has become an increasingly more vibrant task in the field of computer vision research. Even though detecting plant diseases occupies a vital position in practical agricultural production, drawbacks of typical algorithms in deep learning should never be overlooked: (1) The training model requires a high expenditure on hardware, and a massive number of data are necessary. (2) Models are problematic for adapting to practical production due to the low inference speed. (3) Models lack sufficient generalization capability. In addition to the algorithm itself, there are various constraints in detecting plant diseases: (1) The quality of the acquired leaf dataset is influenced by objective factors, such as illumination and leaf growth stage. (2) In an image with multiple leaves, the leaves blocking each other will affect the object detection.

Therefore, this article proposed a Tranvolution detection network with GAN modules, aiming to tackle these mentioned above problems. The following demonstrates primary innovations of the model proposed in this article:

1. GAN modules: First and foremost, a generative model is added in front of the backbone to expand the input leaf images, which aims to alleviate the general problem of small sample size datasets. Second, GAN models are added to the attention extraction module to generate attention masks. Figure 11 shows the effect of adding GAN models on feature maps, and the results of the experimental part also illustrate that this approach can effectively improve the robustness of the model. Ultimately, on the validation set, the proposed method reaches 51.7, 48.1, and 50.3% on *Precision*, *Recall*, and *mAP*, respectively. This experimental result demonstrates that the proposed model outperforms all the comparison models.
2. We modified the Transformer, by reducing the number of parameters, and improving the training speed, to improve CNN's ability to capture global features as a branch network. Because it inherits and combines the structural and global feature extraction advantages of CNN and visual transformers. The performance of Tranvolution is significantly better than CNN and vision transformer with comparable parameter complexity, showing the great potential capability in plant disease detection tasks.
3. In order to verify the effectiveness of various implementations of GAN modules, in Section 7, we validated the performance of different combinations of generative models. The experimental results reveal that the model obtained by the combination of WGAN + SAGAN has the best performance.
4. Based on the model proposed in this article, we optimized it at the command level, deployed it on Hbird E203, and created an intelligent robot that works with actual agricultural scenarios.

Although the proposed model has surpassed the comparison model, limitations still exist. Based on the shortcomings proposed in Section 7.4, the authors of this article will work on redesigning the model's loss function in the future to address the imbalance of the data set and further optimize the model from the perspective of loss function design.

## Data Availability Statement

The original contributions presented in the study are included in the article/supplementary material, further inquiries can be directed to the corresponding author/s.

## Author Contributions

YZ: conceptualization, methodology, validation, visualization, and supervision. YZ, LZ, and SW: writing the original draft preparation. YZ and SW: writing review and editing. CL: project administration and funding acquisition. All authors have read and agreed to the published version of the manuscript.

## Funding

This research was funded by the National Natural Science Foundation of China grant number 61202479.

## Conflict of Interest

The authors declare that the research was conducted in the absence of any commercial or financial relationships that could be construed as a potential conflict of interest.

## Publisher's Note

All claims expressed in this article are solely those of the authors and do not necessarily represent those of their affiliated organizations, or those of the publisher, the editors and the reviewers. Any product that may be evaluated in this article, or claim that may be made by its manufacturer, is not guaranteed or endorsed by the publisher.
